# The Southern Surgical and Gynecological Association

**Published:** 1889-01

**Authors:** 


### THE SOUTHERN SURGICAL AND GYNECOLOGICAL ASSOCIATION.

The first annual meeting of the Association, held in Birmingham, December 4, 5 and 6, was largely attended, and proved a success beyond the most sanguine expectations of even those who had worked hardest in its interest.

It will be seen from the reports of the meeting, that papers were contributed from ten States, and that others were ably represented in the discussions. Five papers were contributed from Georgia; three from Texas; two from Virginia; four from Tennessee; two from Kentucky; four from Mississippi; seven from Alabama; one from Missouri; three from North Carolina, and one from Florida.

The excursions and entertainments arranged by the local committee for the enjoyment of the Association had to be declined, owing to the amount of scientific work on the programme. An example was thus set for future meetings, in declining the many proffered hospitalities of the people of Birmingham, and hereafter the Association will not be expected to devote so much of its time to social pleasures.

Three sessions were held daily, and the members had scarcely time to get their meals. No organization ever did more work in the same time. The members came to work, and were pleased at having the opportunity of devoting the entire time in this way.

There was a motion during the session to limit the membership to one hundred, but it was decided instead of this to make the limit in the high standard of requirements for membership, and not by number. Hereafter every applicant must be endorsed by two members, present an original paper to the Judicial Council, and be recommended by the Council, before his application is submitted to the Association for action. While it was the prevailing opinion that there should not and would not be more than one hundred members, it was decided that the standard adopted would insure this result.

The annual oration was abolished, so as to allow more time for the reading and discussion of papers, notwithstanding the fact that the entertainment afforded by the oration, and the complimentary concert of the Mendelssohn Club on the evening of the first day of the meeting, were greatly enjoyed by the Association and its guests.

This organization is truly a representative body, for its membership consists of the representative surgeons and gynecologists of the entire South, who are deeply interested in its success.

The South can boast of having produced some of the world's most illustrious surgeons and gynecologists, and it affords us great pleasure to see an organization in this section, which will encourage work in these specialties. There can be no question as to the future of the Association.

The following officers were elected: President, Dr. Hunter McGuire, Richmond, Va.; Vice-presidents, Dr. W. O. Roberts, Louisville, Ky., and Dr. Bedford Brown, Alexandria, Va.; Secretary, Dr. W. E. B. Davis, Birmingham; Treasurer, Dr. H. P. Cochrane, Birmingham, Ala.

*Judicial Council:* Dr. John S. Cain, Nashville, Tenn.; Dr. W. T. Briggs, Nashville, Tenn.; Dr. J. M. Taylor, Corinth,

Miss.; Dr. De Saussure Ford, Augusta, Ga., and Dr. Virgil O. Hardon, Atlanta, Ga.

*Publication Committee:* Dr. W. E. B. Davis, Birmingham, Ala.; Dr. W. B. Rogers, Memphis, Tenn., and Dr. Virgil O. Hardon, Atlanta, Ga.

*Chairman of Committee of Arrangements:* Dr. Duncan Eve, Nashville, Tenn.

### ITEMS.

WE wish you a most happy and prosperous New Year.

PROF. JOHN ASHURST, JR., and Prof. Tyson, have been indorsed by the Faculty of the University of Pennsylvania for the chairs of Surgery and Clinical Medicine respectively.

A London surgeon disguised himself and started out on an amateur detective hunt for the Whitechapel murderer. He was promptly detected, set upon by the populace, and only saved by the arrival of the police.

DID you ever figure on how much it costs to run a first-class journal? We have—and paid for it.

DO YOU owe us anything? If so, now is the time to remit. We need money. Your account by itself is small. Several hundred like it count up, and the total is large. Shall we hear from you?

THE Atlanta Society of Medicine held its annual meeting on the evening of December 10th. The following officers were elected for 1889: President, Hunter P. Cooper, M. D.; Vice-President, Wm. Perrin Nicolson, M. D.; Treasurer, E. Van Goidtsnoven, M. D.; Secretary, L. H. Jones, M. D.; Corresponding Secretary and Librarian, N. O. Harris, M. D.; Reporters, E. V. Joye, M. D., Frank O. Stockton, M. D., W. S. Kendrick, M. D.

SIX CHILDREN AT A SINGLE BIRTH.—At Dallas, Texas, on November 3d, Mrs. George Hirsh, of Navarro county, gave birth to six children. The mother and children are doing well. There are four boys and two girls. All are perfect and fully proportioned, but very small. The babies are all tagged to preserve their identity.

PERSONAL—Our publisher, Mr. Herman A. Hasslock, of Hasslock & Ambrose, is a candidate for Public Printer under the incoming administration, and if promptness, energy and a thorough knowledge of the art is a recommendation, he will fill the place with credit to the government and himself. We cordially commend him in every particular, as our relations with him have been close, pleasant and profitable.—*Nashville Journal of Medicine and Surgery*, Dec., 1888.

A CONVENIENT CALENDAR AND STAND.—The most convenient desk calendar for 1889, is the Columbia Bicycle Calendar, issued by the Pope Manufacturing Company, of Boston, Mass., It is in the form of a pad of 365 leaves, 5⅛x2¾ inches, with blanks for memoranda. The leaves are sewed at the ends so that any entire leaf can be exposed whenever desired. The pad so rests upon a portable stand that the entire surface of each leaf is brought directly before the eye. Besides the date and ample room for memoranda, upon each slip appear quotations pertaining to cycling, and about typewriting and stenography, with occasional mention of the new typewriter. Although this is the fourth year of the calendar, the quotations are fresh and new, and the information would, if placed in book type, make a fair sized volume.

A STATE BOARD OF HEALTH FOR GEORGIA.—Dr. Sims, the member of the Legislature from Lincoln, has in course of preparation a bill creating a State Board of Health. The bill will provide for a board consisting of five physicians in good standing, who will have charge of the quarantine regulations of the State, without, however, clashing with the authority of the local boards.

The measure is the result of a general discussion in the Committee on Sanitation and Hygiene, of the recent epidemic in Jacksonville, and of the great variety of quarantine regulations in different parts of the State. One result of the creation of a State Board of Health, will be to make uniform the quarantine regulations. The friends of the measure believe that the presence of a State Board will do much to allay the excitement incident upon epidemics.

EFFECT OF VARNSIHING THE ENTIRE SKIN.—The old story of the sad fate of the gilded child is in danger of being relegated to the limbo of fables, if certain experiments undertaken by a Russian physician, Dr. Tecoutjeff, and recorded in *Wratsch*, are confirmed. According to an abstract published in the *Bulletin General de Therapeutique*, Tecoutjeff has subjected eighteen adults and five children to the test, coating six of them with an irritating ointment, eight with a gelatinous mass, and nine with a mixture of diachylon and lard. They were kept in bed from two to seven days, and five times daily the applications were made to the entire surface, except the head and in some instances the palms of the hands. In none of them were any such serious symptoms noted as are known to take place in the lower animals. The author concludes that smearing the entire skin, at least to any degree ever desirable in therapeutics, is absolutely harmless.—*N. Y. Medical Journal.*

DR. JENNER'S LIFE DEVOTED TO RESIGNING.—A correspondent writes to the *London Pall Mall Gazette:* "Sir William Jenner's resignation is a phase to which the medical profession attach quite an esoteric sense. He has passed his life in resigning. His frequently announced resignation at University College Hospital were among the jokes of the students of the day. He threatened to resign his diploma of the University of London when women were admitted to degrees; he threatened to resign his presidency of the Medical Congress when a similar proposition was made; he resigned the Samaritan Hospital in a pet; and the Children's Hospital. It is said to be the third time he has threatened resignation of the Medical Association. The irony of the present situation is that, according to the whispers current in medical circles, Sir William Jenner set the fashion of furnishing full minute details of the "secrets of the sick chamber" in the information which he furnished for publication in the daily papers and in the medical journals on the occasion of the Prince of Wales' illness. Of this privilege it is unusual to claim a monopoly.—*Ex.*

YELLOW FEVER IN CUBA.—A Washington physician, who is well informed upon West Indian affairs, says an Eastern paper, makes the surprising statement that the native Cubans do not desire to stamp out yellow fever. They suffer but little from it themselves, and are glad to have it kill about 1,000 Spanish soldiers every year. If this is true it is a shocking example of the possible results of race and national prejudice. Cuba is the fever's winter home, and from there every summer it begins a raid upon our Southern cities. It may yet become necessary to annex the island to the United States in order to have it put under proper sanitary condition. As it does not appear to be practicable at present, the best course now open is to take precautions for keeping the infection out of our country next year. Early in the season a rigid quarantine should be established, and the authorities of all Southern cities liable to visitation should see to it that all filth is removed from the streets before it can harbor and propagate the dreaded disease.—*Exchange.*

SURREPTITIOUS ADVERTISING.—In a recent issue of the *New York Press*, we find the following just criticism of a certain class of doctors, who, while professing to be honest, dignified medical men, nevertheless continually bring discredit upon the profession by surreptitiously advertising themselves in the secular papers.

There are more ways of advertising than the manly way of coming to a newspaper office, asking the rates per line and paying cash in advance. Some people think it undignified or unprofessional to advertise in that way, but do not scruple to advertise themselves indirectly without seeming to intend to do so. Of such are the doctors and the undertakers who follow them, somewhat after the manner of night following day, in a celebrated case. Instances of this were seen in the cases of Grant and Garfield, in this country, and in that of Emperor Frederick II., of Germany.

And Indianapolis is not without her quota of such instances. The doctor who cannot refrain from airing his ability and egotism in the secular paper, should have the manhood to step out from among reputable medical men and join the quacks, where he properly belongs.—*Indiana Medical Journal.*

TYPHOID IN VERSE.—At a recent meeting of the New York Academy of Medicine, the following was read:

THE OLD OAKEN BUCKET, AS REVISED AND EDITED BY "A SANITARIAN."

With what anguish of mind I remember my childhood,
Recalled in the light of a knowledge since gained,
The malarious farm, the wet fungus grown wildwood,
The chills then contracted that since have remained;
The scum-covered duck pond, the pig-stye close by it,
The ditch where the sour smelling house drainage fell;
The damp, shaded dwelling, the foul barn-yard nigh it—
But worse than all else was that terrible well,
And the old oaken bucket, the mold crusted bucket,
The moss covered bucket that hung in the well.
Just think of it! Moss on the vessel that lifted
The water I drank in the days called to mind;

Ere I knew what professors and scientists gifted
In the waters of wells by analysis find;
The rotting wood fibre, the oxide of iron,
The algae, the frog of unusual size,
The water impure as the verses of Byron,
Are things I remember with tears in my eyes.
And to tell the sad truth--though I shudder to tell it—
I considered that water uncommonly clear,
And often at noon, when I went there to drink it,
I enjoyed it as much as I now enjoy beer.
How ardent I seized it with hands that were grimy!
And quick to the mud covered bottom it fell!
Then reeking with nitrates and nitrites, and slimy
With matter organic, it rose from the well,
Oh, had I but realized in time to avoid them--
The dangers that lurked in that pestilent draught—
I'd have tested for organic germs, and destroyed them
With potassic permanganate ere I had quaffed,
Or, perhaps, I'd have boiled it and afterward strained it
Through filters of charcoal and gravel combined;
Or, after distilling, condensed and regained it
In potable form, with its filth left behind.
For little I knew of the dread typhoid fever
Which lurked in the water I ventured to drink;
But since I've become a devoted believer
In the teachings of science, I shudder to think,
And now, far removed from the scenes I'm describing,
The story for warning to others I tell,
As memory reverts to my youthful imbibing,
And I gag at the thought of that horrible well,
And the old oaken bucket, the fungus grown bucket—
In fact, the slop bucket—that hung in the well.

J. C. BAYLES.

THE *New York Medical Journal*, November 3, 1888, expresses the opinion that the chewing of gum may be advantageous under certain circumstances, inasmuch as it increases the secretion of saliva, and thus may, if used at the proper time, materially assist the digestion of amylaceous food. It also serves to cleanse the teeth, and may exert a wholesome influence upon the pharynx in catarrhal conditions.

TREATMENT OF ERYSIPELAS.—Nussbaum recommends the application of a pomade of equal parts of lanolin and icthyol as a very speedy and simple method of cure in erysipelas. He covers the parts with salicylated cotton, and obtains a painless cure usually in two or three days.

SUSPECTED A TRAP.—"Here's an article headed, 'Marvelous Escape of a Distinguished Citizen from a Horrible Death,'" said the dutiful daughter, who was reading the morning paper to her invalid father. "The friends of Mr. J. Alpheus Bramble were shocked on learning, a few mornings ago, that—" "Jane," interrupted the irritable parent, "before you read any more of that you will oblige me if you'll look about half way down to the bottom of the article and see whose patent medicine it's advertising."

WHAT CONSTITUTES A MEDICAMENT?—A chemist of Fleet street was lately summoned for selling patent medicines, to-wit, "corn solvents," without the necessary government stamp. It was contended for the defence that corn solvents and such like things were not contemplated by the Act of George II, under which the proceedings were taken. A corn was not an "ailment of the body." Mr. Bridge: How about toothache? Mr. Rose-Innes, who appeared for the defendant, was bound to admit, from personal experience, that that was an ailment. Mr. Bridge decided that this and other articles sold by the defendant came within the statute, and imposed a penalty of £5 and costs 8s. In two or three cases he held that the charge had not been proved—such as, for instance, when concentrated essence of ginger was sold, and another where a compound was described as a toothpaste for beautifying the gums and preventing toothache. Pears' soap was said to beautify the skin. Preventing a disease was different from curing one. A tooth tincture for curing toothache came under the word "medicament."—*British Medical Journal.*

